# Depression and Associated Factors in Chinese Patients With Chronic Kidney Disease Without Dialysis: A Cross-Sectional Study

**DOI:** 10.3389/fpubh.2021.605651

**Published:** 2021-05-28

**Authors:** Difei Duan, Lin Yang, Min Zhang, Xiaoli Song, Wen Ren

**Affiliations:** ^1^Department of Nephrology, West China Hospital, Sichuan University, Sichuan, China; ^2^School of Nursing, The Hong Kong Polytechnic University, Hong Kong, China; ^3^West China School of Nursing, Sichuan University, Sichuan, China

**Keywords:** depression, illness perception, pain perception, self-esteem, chronic kidney disease

## Abstract

**Background:** Chronic kidney disease (CKD) has been a globally public health problem over the past decades. The maintenance of physical and mental health is of importance for patients nowadays. Notably, depression is prevalent and associated with various adverse events in CKD patients without dialysis. Prior studies have reported that pain, negative illness perception, pain, and low self-esteem are potential risk factors of depression, while few studies have comprehensively investigated the mechanisms among these factors and depression among this population.

**Purpose:** This study aims to investigate the prevalence of depression and further explore the factors associated with depression among CKD patients without dialysis in China.

**Design and Methods:** We conducted a cross-sectional study in patients with diagnosed CKD to investigate the prevalence of depression was by the Beck Depression Inventory-II (BDI-II). The data on pain interference, illness perception, and self-esteem were also collected via self-administered questionnaires. A structural equation model (SEM) was used to examine the factors associated with depression.

**Main Findings:** From June to October 2019, we successfully interviewed 334 CKD patients at the outpatient clinics. Their mean age was 45.6 years (ranging from 19 to 74 years), and 48.5% were male. Most respondents were at early CKD stages (77.5% stage 1–3) and the prevalence of depression was 22.2%. We found a moderate association between illness perception and depression, which was modified by self-esteem. Similar but weaker association was found between pain interference and depression.

**Conclusion and Recommendations:** Negative illness perception, low self-esteem and severe pain interference were associated with depression among Chinese CKD patients without dialysis. Future studies are warranted to investigate the underlying mechanism and formulate the intervention strategies for this high-risk population.

## Introduction

Chronic kidney disease (CKD) is characterized by progressive loss of renal function and classified as five stages based on estimated glomerular filtration rate (eGFR) according to Kidney Disease: Improving Global Outcomes (KDIGO) guidelines (1). CKD is a global public health problem (2); the all-age mortality rate of CKD increased worldwide by 41.5% (95% uncertainty interval: 35.2–46.5) between 1990 and 2017 (3). A national cross-sectional survey conducted in 2012 showed that the prevalence of CKD among Chinese adults was 10.8% (95% confidence interval: 10.2–11.3) (4). CKD is irreversible; when it progresses to stage 5, patients experience kidney failure that requires replacement therapy (e.g., dialysis treatment) to prolong life. However, in 2016, the annual mortality rate of patients with hemodialysis in Sichuan Province, China was 58.39% person-years (5). Effective management including early initiation of dialysis treatment is critical for delaying the progression of CKD (6).

Multidisciplinary management strategies have become commonly used to promote both the physical and mental well-being of patients (7). One of the most common mental illnesses in CKD patients without dialysis was depression (8), which was typically diagnosed through interviews and self-report questionnaires. Depression was associated with low treatment adherence, early initiation of dialysis, and high risk of hospitalization and death in CKD patients without dialysis (9, 10). The prevalence of depressive symptoms in CKD patients at stage 1–5 was as a high as 26.5% (11). Many studies have focused on the negative impact of depressive symptoms on the outcome of CKD. Previous studies in Western populations have shown that negative illness perception, pain, and low self-esteem are potential risk factors for depression (12–14); however, the factors associated with depression in CKD patients remain poorly understood. Some studies have adopted behavioral models to address this question (15). The Common Sense Self-Regulation Model (CS-SRM) proposed that individual perception of illness and the coping strategies used—but not the disease itself—determined behavioral and emotional responses such as depression. The CS-SRM has been successfully applied to predict the progression of different chronic diseases, including diabetes, hypertension, and cancer (16). However, there were limited data on the contribution of illness perception to depression in CKD patients without dialysis (17). One study of 80 CKD patients (36% without and 64% with dialysis) found that negative illness perception significantly increased the incidence of depression (18) but only 29 patients at stage 3–5 were recruited, which limits the generalizability of this finding.

It has been well-recognized that pain had a negative impact on illness perception and self-esteem (12, 19, 20). Most CKD patients experienced chronic, acute, or paroxysmal pain that were caused by the disease itself and underlying comorbidities (21). Despite the high prevalence, pain in CKD patients has been under-recognized and its severity was underestimated by physicians in renal clinics (22), partially due to the lack of comprehensive assessment tools. pain interference measures the extent to which physical pain impeded daily life and well-being, (23) and is an important aspect of pain assessment in addition to pain intensity, (24). Pain intensity was shown to be positively associated with depressive symptoms in CKD patients (25). However, there is limited data on the relationship between pain interference and illness perception and self-esteem (26, 27).

Self-esteem is based on positive and negative feelings that an individual has about him/herself, which create a sense of self-worth. Self-esteem is both an outcome and determinant of health behavior, and thus plays an important role in chronic illness management (28). In particular, high self-esteem is a resource for coping with disease and serves as a buffer against stress and depression (29). However, living with chronic diseases might lower patients' self-esteem (30). As their disease progresses, CKD patients may experience lower self-esteem as a result of anxiety and feelings of desperation regarding their prognosis and the effectiveness of treatments, which can lead to depression (31). In a survey among 109 CKD patients at stage 4 or 5, lower self-esteem was found to be associated with higher illness perception (32), and higher risk of depression in young adults with end-stage CKD (33). However, the role of self-esteem has not been investigated in CKD patients without dialysis. Moreover, most studies were conducted in Western countries, which might not be generalized to Chinese populations.

**Figure 1:**
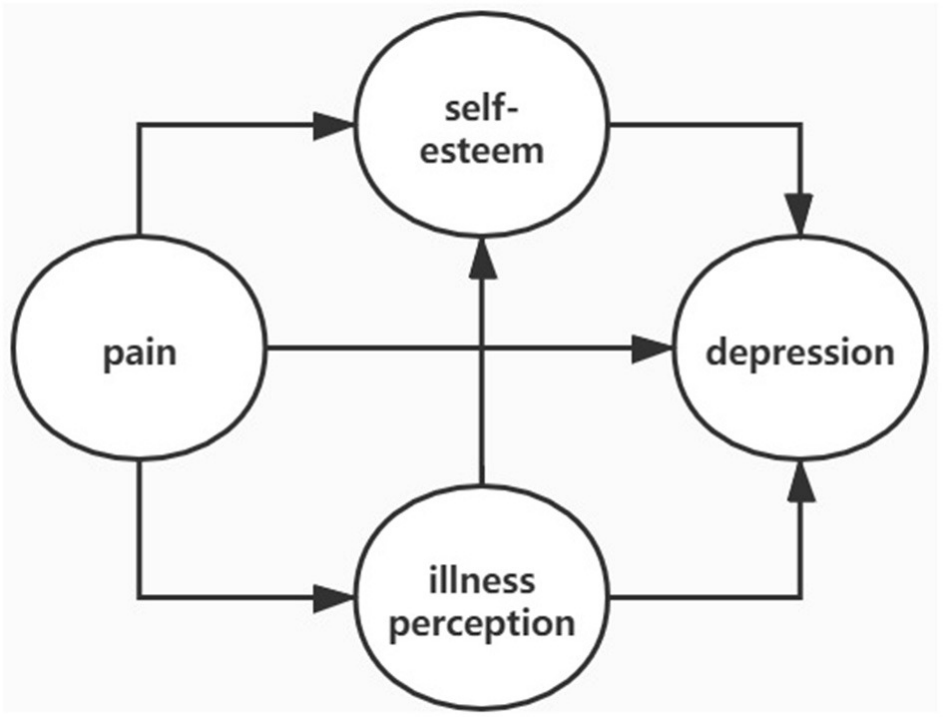
Theoretical framework for the development of depression in CKD patients.

To date there have been no studies investigating the association between pain interference, illness perception, self-esteem, and depression in CKD patients without dialysis. This is due in part to the fact that Chinese patients avoid seeking medical assistance for mental illness and because mental healthcare is seldom included in the management of chronic diseases (34). In the present study, we tested the hypothesis that severe pain interference is associated with negative illness perception and low self-esteem in CKD patients without dialysis, leading to depression (Figure 1).

## Materials and Methods

### Study Design and Participants

This cross-sectional study was conducted in a large tertiary hospital (>4,000 beds) in Southwestern China. The research assistants were nurses in the Department of Nephrology who were trained on the standardized procedures for collecting information *via* questionnaires. Participants were invited to complete the self-administered questionnaire while they attended the renal clinic after they had signed the consent form. The completion time for the questionnaire was 15–20 min. The inclusion criteria were as follows: (1) age ≥18 years; (2) diagnosed with CKD by a nephrology specialist; (3) under regular follow-up at the renal clinic; and (4) able to speak Mandarin. The exclusion criteria were as follows: (1) diagnosed with a life-threatening condition (e.g., acute respiratory distress syndrome, acute heart failure, etc.); (2) receiving dialysis treatments or received kidney transplantation surgery; (3) unable to complete the survey; and (4) previously diagnosed with depression or received depression treatment. According to KDIGO guidelines, CKD patients were classified as stage 1 (eGFR ≥ 90 ml/min/1.73 m^2^), stage 2 (eGFR = 60–89 ml/min/1.73 m^2^), stage 3 (eGFR = 30–59 ml/min/1.73 m^2^), stage 4 (eGFR = 15–29 ml/min/1.73 m^2^), or stage 5 (eGFR <15 ml/min/1.73 m^2^).

### Sample Size

The sample size was primarily calculated with the aim of investigating the prevalence of depression in the cohort [26.5% in CKD stage 1–5 patients (11)]. With an α of 0.05, 2-tailed testing, power of 0.80, and margin of error of 6%, we estimated that 334 participants needed to be recruited. In line with the rule-of-thumb of 10 cases per variable, the number of participants was deemed sufficient for the structural equation model (SEM).

### Outcome Measurement

Depression was evaluated with the Beck Depression Inventory-II (BDI-II), a 21-item questionnaire with total score ranging from 0–63 (35) and each item scored from 0–3 on a Likert scale. The BDI-II has satisfactory internal consistency and reliability for assessing CKD patients without dialysis (36). In this study, depression was defined as a BDI-II score ≥11 (37). The Cronbach's α was 0.86. Based on the factor structure of BDI-II (38), the 21 items were parceled as 3 components and used as indicators of latent depression variables (somatic, cognitive, and affective observed items) (39).

### Other Instruments

Pain interference was measured with the Chinese version of the Brief Pain Inventory (BPI) (40), which has seven items, each scored from 0–10, that assess the degree to which pain has interfered with daily living in the 3 months prior to the assessment. The recall period of pain assessment was shortened to 1 month as in a previous study (41). Participants were asked whether their pain had persisted for >3 months to screen out those suffering from chronic pain. The Cronbach's α of pain interference was 0.91. Confirmatory factor analysis (CFA) was performed and three items were retained with a goodness-of-fit for this measurement model (42): the extent to which pain interferes with general activities (Item 1), sleep quality (Item 6), and enjoyment of life (Item 7).

Illness perception was assessed with the Chinese version of the Brief Illness Perception Questionnaire, which comprises eught items scored from 0 (strongly disagree) to 10 (strongly agree) that assess cognitive and emotional representations of illnesses (43). A higher total score indicates a more negative illness perception. The Cronbach's α was 0.70. We used CFA to combine the eight items into three items: the extent to which the illness affects daily life (Item 1), concern about the illness (Item 6), and emotions (Item 8).

Self-esteem was assessed with the Rosenberg Self-Esteem Scale, which has 10 items each scored from 1 (strongly agree) to 4 (strongly disagree). A higher score represents higher self-esteem. In previous studies, the scale has shown good reliability in CKD patients (44). The Cronbach's α was 0.87. Four items were identified from the CFA for self-esteem: “I feel that I'm a person of worth, at least on an equal plane with others” (Item 1); “I feel that I have a number of good qualities” (Item 2); “I am able to do things as well as most other people” (Item 3); and “Overall I am satisfied with myself” (Item 7).

Demographic data (age, sex, income, marital status, etc.) and clinical data (comorbidities, CKD stage, time since CKD diagnosis) were collected from self-report questionnaires or electronic medical records at the hospital.

### Data Analysis

Descriptive statistics such as mean and standard deviation were applied to continuous variables, whereas frequency and percentage were used for categorical data. Univariate linear regression was performed to select the variables with *p*-value < 0.1 which were included in the subsequent multivariate linear regression analysis. The significant (*p* < 0.05) variables were entered into the SEM for further analysis.

We removed outliers using the Mahalanobis distance test and further assessed data normality. The remaining data (*n* = 326) were used for the SEM with maximum likelihood estimation. CFA was performed to determine whether the goodness-of-fit of the measurement model was satisfactory. Multifactor CFA was carried out to evaluate the discriminant validity of variables included in the final model (45). For each pair of latent variables, average variance extracted (AVE) exceeding the square of the correlation coefficient indicated that the pair was sufficiently different to be recognized as separate variables. The final model was derived by adding pathways or removing variables while inspecting changes in goodness-of-fit indices and standardized residuals. The goodness-of-fit of the structural model was measured by the Chi square test, standardized root-mean-square residual (SRMR), root-mean-square error of approximation (RMSEA), adjusted goodness-of-fit (AGFI), comparative fit index (CFI), goodness-of-fit (GFI), and Tucker Lewis index (TLI) (46). After testing for multivariate normal distribution, the data were bootstrapped 2,000 times to increase the goodness-of-fit of the model (47). All statistical analyses were performed using SPSS v25.0, and SEM was performed using Amos v24.0 (both from IBM, Armonk, NY, USA).

## Results

### Study Population

Between June and October 2019, 334 CKD participated in the study for a response rate of 45%. The demographic characteristics and disease history of the study population are shown in Table 1. The mean age was 45.6 years (range: 19–74 years), and 48.5% were male. Most (>75%) were in an early stage of CKD and the average time since CKD diagnosis was 24 months. Comorbidities including hypertension and diabetes were present in 48.8% of participants. A total of 74 patients (22.2%) were diagnosed with depression (BDI-II score >11).

**Table 1:** Demographic and clinical data of CKD patients.

**Characteristic**	***n* (%)**	
Number of participants	334	
**Sex**, ***n*** **(%)**		
Male	158	(47.3)
Female	176	(52.7)
Mean age, years (SD)	45.6	(12.754)
Range	19–73	
**Occupation**, ***n*** **(%)**		
Employed	260	(77.8)
Unemployed	74	(22.2)
**Education**, ***n*** **(%)**		
Primary or below	58	(17.4)
Junior or high school	166	(49.7)
College or above	110	(32.9)
**Marital status**, ***n*** **(%)**		
Married	276	(82.6)
Single	36	(10.8)
Other	22	(6.6)
**Place of residence**, ***n*** **(%)**		
Urban	201	(60.2)
Suburban	98	(29.3)
Village	35	(10.5)
**Monthly personal income (RMB)**, ***n*** **(%)**		
<2,000 or no income	102	(30.5)
2,000–5,000	94	(28.1)
5,000–≥8,000	64	(19.2)
Not reported (unknown)	71	(21.3)
**History of smoking**, ***n*** **(%)**		
Current	40	(12.0)
Ex-smoker	48	(14.4)
Never	246	(73.7)
**History of alcohol drinking**, ***n*** **(%)**		
Current	36	(10.8)
Ex-drinker	223	(66.8)
Never	75	(22.5)
**CKD stage**, ***n*** **(%)**		
G1	80	(24.0)
G2	82	(24.6)
G3	97	(29.0)
G4	51	(15.3)
G5	24	(7.2)
Months since CKD diagnosis, median (IQR)	24	(50.0)
**Comorbidity**, ***n*** **(%)**		
Hypertension	84	(25.1)
Diabetes	43	(12.9)
Both	17	(5.1)
Other	19	(5.7)
None	171	(51.2)
**Depression**		
Yes	74	(22.2)
No	260	(77.8)
**Pain characteristic**		
Chronic	109	(32.6)
Other type	69	(20.7)
None	156	(46.7)

*CKD, chronic kidney disease; IQR, interquartile range; RMB, renminbi*.

### Association Between Pain, Illness Perception, Self-Esteem, and Depression in CKD Patients Without Dialysis

Pain interference was positively associated with illness perception and depression and negatively associated with self-esteem (Table 2). Illness perception was positively associated with depression and negatively associated with self-esteem. Self-esteem was negatively associated with depression.

**Table 2:** Sample correlations and mean (SD) of the main variables (*n* = 334).

	**Depression**	**Illness perception**	**Self-esteem**	**Mean (SD)**
Depression	1			5.86 (5.84)
Illness perception	0.54^**^			34.48 (11.59)
Self-esteem	−0.41^**^	−0.59^**^		30.16 (4.50)
Pain interference	0.44^**^	0.36^**^	−0.27^**^	1.21 (1.80)

** *P < 0.01*.

*SD, standard deviation*.

Regarding the measurement model, the scales showed good convergent and discrimination validity, with AVE>0.5 and construct reliability >0.7 (48) (Table 3).

**Table 3:** Reliability and validity of instruments for assessment of pain, illness perception, self-esteem, and depression (*n* = 326).

**Latent variable**	**Item**	**Convergent validity**				
		***P*-value**	**Factor loading**	**Item reliability**	**CR**	**AVE**
Pain	P1		0.887	0.787	0.930	0.815
	P2	<0.001	0.893	0.797		
	P3	<0.001	0.928	0.861		
Self-esteem	R1		0.794	0.630	0.875	0.636
	R2	<0.001	0.783	0.613		
	R3	<0.001	0.851	0.724		
	R4	<0.001	0.759	0.576		
Depression	DP1		0.864	0.746	0.849	0.655
	DP2	<0.001	0.686	0.471		
	DP3	<0.001	0.864	0.746		
Illness perception	I1		0.738	0.545	0.883	0.718
	I2	<0.001	0.860	0.740		
	I3	<0.001	0.932	0.869		

*AVE, average variance extracted; CR, construct reliability*.

**Figure 2:**
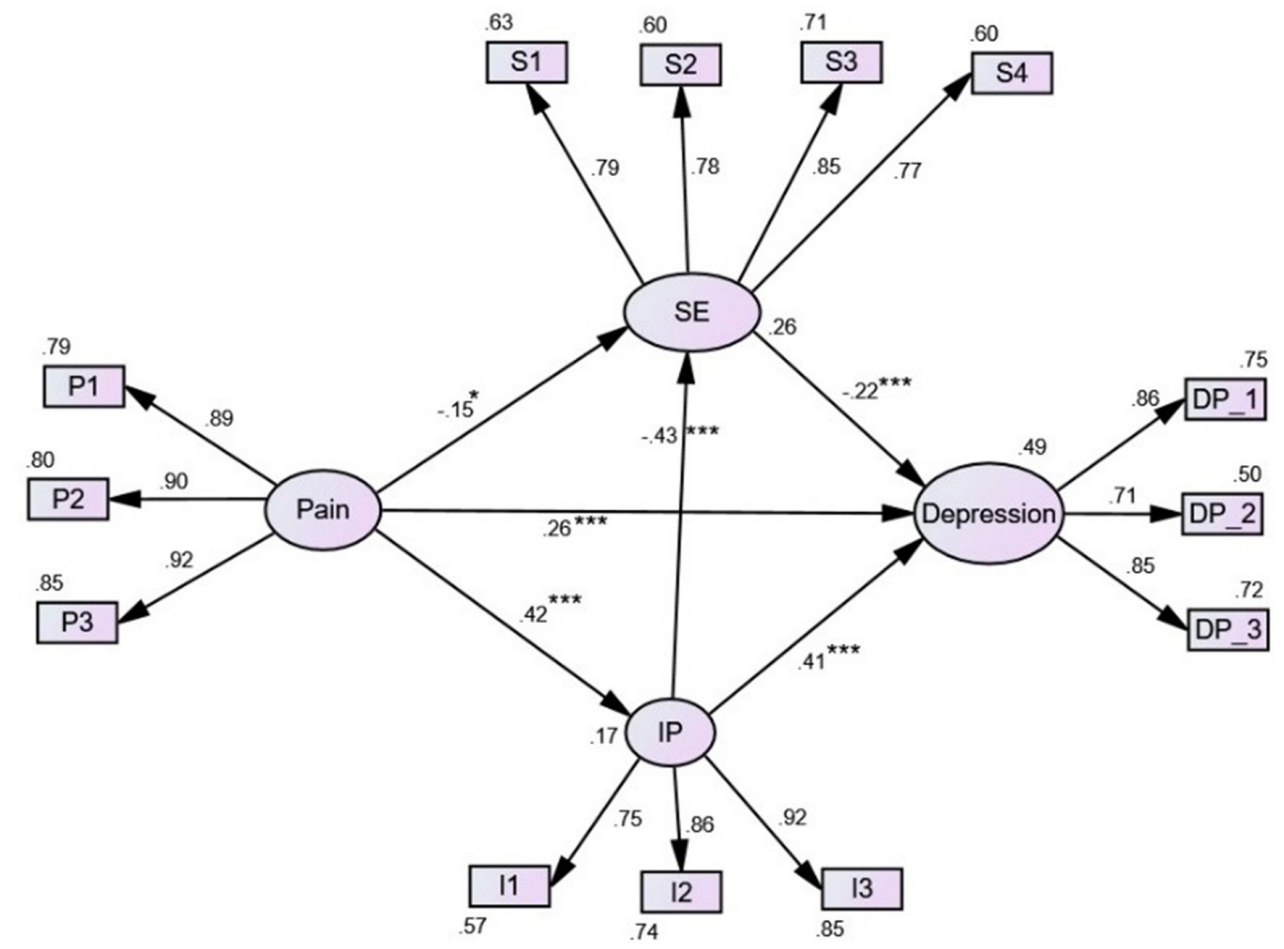
Diagram of the SEM. IP, illness perception; SE, self-esteem. *p < 0.05, ***p < 0.001. All parameters are standardized regression weights.

As the demographic and clinical variables were not significantly associated with depression in the multivariate regression analysis, only pain interference, self-esteem, illness perception, and depression were entered into the SEM analysis. Overall, the final model had satisfactory goodness-of-fit (X2 = 69.219; X2/df = 1.17; GFI = 0.095; AGFI = 0.951; CFI = 0.985; TLI = 0.982; RMSEA = 0.030; SRMR = 0.043) (Figure 2). Pain interference and illness perception accounted for 26% of the self-esteem variance, and pain interference alone explained 17% of the illness perception variance. Pain interference, illness perception, and self-esteem together accounted for 49% of the variance in depression. Pain interference and illness perception showed a significant positive association with depression (β = 0.26 and 0.41, *p* < 0.001), whereas a negative association was found for self-esteem (β = −0.22, *p* < 0.001). Illness perception (β = −0.43, *p* < 0.001) and pain (β = −0.15, *p* = 0.012) were negatively associated with self-esteem, and pain was positively associated with illness perception (β = 0.42, *p* < 0.001).

## Discussion

Depression has adverse health effects in CKD patients without dialysis (9), resulting from treatment non-adherence and unhealthy lifestyle (49). Systemic inflammation in depression can also lead to suppression of patients' immune system (50), which can accelerate disease progression. In our study, the prevalence of depression in stage 1–5 CKD patients was 22.2%, which is comparable to the rate of 20.6–26.5% reported by others (11, 51). We did not find significant differences in depression rates across different CKD stages, in accordance with earlier findings in stage 2–5 CKD outpatients (52). However, some studies have reported that patients with stage 4 or 5 CKD are more likely to have depression than those in early stages of the disease (51, 53). These inconsistent findings may be attributable to the BPI scale used in our study, which can effectively screen out depression without impacting disease severity (advanced CKD stage or comorbidities) (36). Additional studies with a larger sample of patients at different stages of CKD are needed to clarify the development of depression in CKD. Moreover, taking into account the high prevalence of depression, the assessment of mental health in CKD patients should not be overlooked or underestimated by nephrologists. Compared to Western counties, there are many barriers to mental healthcare that must be overcome in China; particularly challenging are the under-recognition of the need for treatment, concerns over taking psychiatric medications, and uncertainty over healthcare workers' roles (34). Collectively, these factors increase the risk of depression in Chinese patients with CKD.

Depression is frequently overlooked because it often coexists with pain (54). Pain symptoms can delay the diagnosis of depression, potentially leading to more serious depression and worse outcomes for patients (55). However, the complexity of pain experienced by CKD patients makes comprehensive pain assessment difficult. We found that pain interference was positively associated with depression, which is consistent with previous studies of hemodialysis patients (56). It was recently reported that pain interference was more significantly associated with depression than pain intensity in cohorts with various pain issues (57, 58), implying that with the appropriate assessment tool, pain interference can provide more useful information for healthcare workers. More specifically, patient-reported outcome measures of pain interference can help healthcare workers design personalized illness or symptom management programs (59). In agreement with other studies (20, 26, 60), we found that pain had a negative impact on CKD patients' self-esteem and was associated with negative illness perceptions. Conversely, individuals without pain problems are more likely to have positive feelings and a higher sense of self-worth than those with pain issues (60). Given that illness perception can be affected by somatic symptoms such as pain (61), we speculate that pain can exacerbate worries about disease progression in CKD patients.

Negative illness perception in CKD patients was shown to be associated with depression either directly or indirectly through self-esteem (18, 32). Our results supported these findings: as in previous investigations of patients with chronic illnesses (62), a more negative perception of the illness was associated with lower self-esteem. We speculate that these CKD patients recognized the chronicity of their disease and were increasingly required to cope with illness-related problems as the disease progressed (30). In the process, their social and family roles may have diminished, further decreasing their self-esteem. Patients at advanced stages of CKD may also have concerns on the safety and effectiveness of treatments (63). As a key aspect of disease adaption in CKD patients (17), negative illness perception could contribute to the development of depressive symptoms. According to self-regulation theory, illness perception is modifiable (64), implying that regular assessment of illness perception and early intervention for depression may be beneficial for CKD patients. We also found that the variance of illness perception was not fully explained; therefore, further research is needed to identify the factors influencing illness perception in early-stage CKD patients.

The health benefits of high self-esteem have been demonstrated in CKD patients. For example, higher self-esteem was associated with greater self-efficacy and social support, which could prevent the onset or progression of CKD (65). Although the association between depression and low self-esteem is well-established, ours is the first study to report a link between self-esteem and depression in Chinese CKD patients without dialysis. It is worth noting that low self-esteem not only predicted depression, but was also a risk factor for non-adherence to depression treatment (29). Self-esteem can be modified by health behavior interventions (66), hence incorporating screening for low self-esteem into CKD management programs could reduce the risk of depression in patients. Given that the small-to-moderate variance of self-esteem was explained in our study and considering the findings of an earlier report (32), self-esteem may be more closely related to illness perception than pain interference. The association between pain and self-esteem warrants further study.

The diagnosis and treatment of depression in CKD patients are clinically challenging, in part because of physicians' concerns about the efficacy and safety of pharmacologic treatments (8). A similar issue exists in the treatment of pain in CKD patients; many nephrologists do not have confidence in existing pain medications (22) given the renal toxicity of conventional analgesics (67). Cognitive behavioral therapy—the most commonly used and effective evidence-based psychotherapy for the treatment of depression in individuals with chronic illnesses (68)—may therefore be particularly beneficial for this population (15). Our study identified relevant variables (e.g., self-esteem and illness perception) associated with pain and depression that can be modified through intervention strategies.

There were some limitations in our study. Firstly, the cross-sectional design did not allow us to establish a causal relationship between the examined variables. Longitudinal studies are warranted to identify the risk factors for depression development in CKD patients. Nonetheless, we attempted to explain the relationship between these variables based on the classic CS-SRM theory. Secondly, the participants were recruited from a single medical center in China, and the relatively small sample size limits the generalizability of the findings. Thirdly, the prevalence of depression was assessed by using a self-report questionnaire less accurate than clinical interviews. Last but not least, although we considered several confounding factors in the model, there could be still some factors that remain unadjusted.

## Conclusion

In conclusion, we found that depression was common in CKD patients without dialysis in China, and was associated with patients' perception of their illness. Our findings provide insight into the associated factors for depression in CKD patients. Future longitudinal studies could further assess the causal effects of these modifiable risk factors for depression, in order to develop intervention strategies to improve patients' quality of life and clinical outcomes.

## Data Availability Statement

The datasets presented in this article are not readily available because due to the nature of this research, the hospital of this study did not agree for their data to be shared publicly, so supporting data is not available. Requests to access the datasets should be directed to Difei Duan, duandifei89@163.com.

## Ethics Statement

The studies involving human participants were reviewed and approved by the Research Ethics Committee of The Hong Kong Polytechnic University. West China Hospital of Sichuan University Biomedical Research Ethics Committee. The patients/participants provided their written informed consent to participate in this study.

## Author Contributions

LY and DD: conceptualization, methodology, and writing review and editing. MZ, XS, and WR: investigation. DD and MZ: resource. DD: formal data analysis and writing-original draft. LY: supervision. All authors contributed to the article and approved the submitted version.

## Conflict of Interest

The authors declare that the research was conducted in the absence of any commercial or financial relationships that could be construed as a potential conflict of interest.

## References

[1] Levey AS Eckardt KU Dorman NM Christiansen SL Hoorn EJ Ingelfinger J. Nomenclature for kidney function and disease: report of a kidney disease: improving global outcomes (KDIGO) consensus conference. Kidney Int. (2020) 97:1117–29. 10.1016/j.kint.2020.02.010 32409237

[2] GBD 2015 Mortality and Causes of Death Collaborators. Global, regional, and national life expectancy, all-cause mortality, and cause-specific mortality for 249 causes of death, 1980–2015: a systematic analysis for the Global Burden of Disease Study 2015. Lancet. (2016) 388:1459–544. 10.1016/S.0140-6736(16)31012-1 27733281 PMC5388903

[3] GBD Chronic Kidney Disease Collaboration Global regional and national burden of chronic kidney disease 1990–2017: a systematic analysis for the Global Burden of Disease Study 2017. Lancet. (2020) 395:709–33. 10.1016/s0140-6736(20)30045-3 32061315 PMC7049905

[4] Zhang L Wang F Wang L Wang W Liu B Liu J. Prevalence of chronic kidney disease in China: a cross-sectional survey. Lancet. (2012) 379:815–22. 10.1016/S0140-6736(12)60033-6 22386035

[5] Zou Y Hong D He Q Wen Y Li G. Epidemiology investigation and analysis of patients with hemodialysis in Sichuan province of China. Ren Fail. (2019) 41:644–9. 10.1080/0886022X.2019.1612429 31296088 PMC6691842

[6] Liyanage T Ninomiya T Jha V Neal B Patrice HM Okpechi I. Worldwide access to treatment for end-stage kidney disease: a systematic review. Lancet. (2015) 385:1975–82. 10.1016/S0140-6736(14)61601-9 25777665

[7] Chen TK Knicely DH Grams ME. Chronic kidney disease diagnosis and management: a review. JAMA. (2019) 322:1294–304. 10.1001/jama.2019.14745 31573641 PMC7015670

[8] Bautovich A Katz I Smith M Loo CK Harvey SB. Depression and chronic kidney disease: a review for clinicians. Aust N Z J Psychiatry. (2014) 48:530–41. 10.1177/0004867414528589 24658294

[9] Chiang HH Guo HR Livneh H Lu MC Yen ML Tsai TY. Increased risk of progression to dialysis or death in CKD patients with depressive symptoms: a prospective 3-year follow-up cohort study. J Psychosom Res. (2015) 79:228–32. 10.1016/j.jpsychores.2015.01.009 25659439

[10] Tsai YC Chiu YW Hung CC Hwang SJ Tsai JC Wang SL. Association of symptoms of depression with progression of CKD. Am J Kidney Dis. (2012) 60:54–61. 10.1053/j.ajkd.2012.02.325 22495469

[11] Palmer S Vecchio M Craig JC Tonelli M Johnson DW Nicolucci A. Prevalence of depression in chronic kidney disease: systematic review and meta-analysis of observational studies. Kidney Int. (2013) 84:179–91. 10.1038/ki.2013.77 23486521

[12] IsHak WW Wen RY Naghdechi L Vanle B Dang J Knosp M. Pain and depression: a systematic review. Harv Rev Psychiatry. (2018) 26:352–63. 10.1097/hrp.0000000000000198 30407234

[13] Järemo P Arman M Gerdle B Larsson B Gottberg K. Illness beliefs among patients with chronic widespread pain—associations with self-reported health status, anxiety and depressive symptoms and impact of pain. BMC Psychol. (2017) 5:24. 10.1186/s40359-017-0192-1 28679446 PMC5499007

[14] Rieger S Göllner R Trautwein U Roberts BW. Low self-esteem prospectively predicts depression in the transition to young adulthood: a replication of orth, robins, roberts (2008). J Pers Soc Psychol. (2016) 110:e16–e22. 10.1037/pspp0000037 25915130

[15] Shirazian S Grant CD Aina O Mattana J Khorassani F Ricardo AC. Depression in chronic kidney disease and end-stage renal disease: similarities and differences in diagnosis, epidemiology, and management. Kidney Int Rep. (2016) 2:94–107. 10.1016/j.ekir.2016.09.005 29318209 PMC5720531

[16] Hagger MS Koch S Chatzisarantis N Orbell S. The common sense model of self-regulation: meta-analysis and test of a process model. Psychol Bull. (2017) 143:1117–54. 10.1037/bul0000118 28805401

[17] Muscat P Chilcot J Weinman J Hudson J. Exploring the relationship between illness perceptions and depression in patients with chronic kidney disease: a systematic literature review. J Ren Care. (2018) 44:174–85. 10.1111/jorc.12243 29806175

[18] Knowles S Swan L Salzberg M Castle D Langham R. Exploring the relationships between health status, illness perceptions, coping strategies and psychological morbidity in a chronic kidney disease cohort. Am J Med Sci. (2014) 348:271–6. 10.1097/MAJ.0000000000000242 24751421

[19] de Raaij EJ Ostelo RW Maissan F Mollema J Wittink H. The association of illness perception and prognosis for pain and physical function in patients with noncancer musculoskeletal pain: a systematic literature review. J Orthop Sports Phys Ther. (2018) 48:789–800. 10.2519/jospt.2018.8072 29747539

[20] Nagyova I Stewart RE Macejova Z van Dijk JP van WJ. The impact of pain on psychological well-being in rheumatoid arthritis: the mediating effects of self-esteem and adjustment to disease. Patient Educ Couns. (2005) 58:55–62. 10.1016/j.pec.2004.06.011 15950837

[21] Cohen SD Davison SN Kimmel PL. Chapter 78 - pain and chronic kidney disease. In: Kimmel PL Rosenberg ME, editors. Chronic Renal Disease. 2nd ed. Cambridge: Academic Press (2020). p. 1279–89. 10.1016/B978-0-12-815876-0.00078-4

[22] Koncicki HM Unruh M Schell JO. Pain management in CKD: A guide for nephrology providers. Am J Kidney Dis. (2017) 69:451–60. 10.1053/j.ajkd.2016.08.039 27881247

[23] Thomas E Peat G Harris L Wilkie R Croft PR. The prevalence of pain and pain interference in a general population of older adults: cross-sectional findings from the north staffordshire osteoarthritis project (NorStOP). Pain. (2004) 110:361–8. 10.1016/j.pain.2004.04.017 15275787

[24] Wilson M. Integrating the concept of pain interference into pain management. Pain Manag Nurs. (2014) 15:499–505. 10.1016/j.pmn.2011.06.004 24882027

[25] Cohen SD Patel SS Khetpal P Peterson RA Kimmel PL. Pain, sleep disturbance, and quality of life in patients with chronic kidney disease. Clin J Am Soc Nephrol. (2007) 2:919–25. 10.2215/CJN.00820207 17702733

[26] Chisari C Chilcot J. The experience of pain severity and pain interference in vulvodynia patients: the role of cognitive-behavioural factors, psychological distress and fatigue. J Psychosom Res. (2017) 93:83–9. 10.1016/j.jpsychores.2016.12.010 28107898

[27] Hegarty D Wall M. Prevalence of stigmatization and poor self-esteem in chronic pain patients. J Pain Relief. (2014) 3:136. 10.4172/2167-0846.1000136

[28] Cotter VT Gonzalez EW Fisher K Richards KC. Influence of hope, social support, and self-esteem in early stage dementia. Dementia. (2018) 17:214–24. 10.1177/1471301217741744 29164906

[29] Stein-Shvachman I Karpas DS Werner P. Depression treatment non-adherence and its psychosocial predictors: differences between young and older adults? Aging Dis. (2013) 4:329–36. 10.14336/ad.2013.0400329 24307966 PMC3843650

[30] Bonsaksen T Fagermoen MS Lerdal A. Factors associated with self-esteem in persons with morbid obesity and in persons with chronic obstructive pulmonary disease: a cross-sectional study. Psychol Health Med. (2015) 20:431–42. 10.1080/13548506.2014.959529 25220791

[31] Kerklaan J Hannan E Hanson C Guha C Cho Y Christian M. Perspectives on life participation by young adults with chronic kidney disease: an interview study. BMJ Open. (2020) 10:e037840. 10.1136/bmjopen-2020-037840 33067282 PMC7569939

[32] Jansen DL Grootendorst DC Rijken M Heijmans M Kaptein AA Boeschoten EW. Pre-dialysis patients' perceived autonomy, self-esteem and labor participation: associations with illness perceptions and treatment perceptions. A cross-sectional study. BMC Nephrol. (2010) 11:35. 10.1186/1471-2369-11-35 21138597 PMC3019121

[33] Murray PD Dobbels F Lonsdale DC Harden PN. Impact of end-stage kidney disease on academic achievement and employment in young adults: A mixed methods study. J Adolesc Health. (2014) 55:505–12. 10.1016/j.jadohealth.2014.03.017 24845867

[34] Sun KS Lam TP Wu D. Chinese perspectives on primary care for common mental disorders: barriers and policy implications. Int J Soc Psychiatry. (2018) 64:417–26. 10.1177/0020764018776347 29781372

[35] Beck AT Steer RA Brown GK. anual for the Beck Depression Inventory-II. San Antonio, TX: Psychological Corporation (1996).

[36] Toups M Carmody T Trivedi MH Rush AJ Hedayati SS. Performance of depression rating scales in patients with chronic kidney disease: An item response theory-based analysis. General Hosp Psychiatry. (2016) 42:60–6. 10.1016/j.genhosppsych.2016.07.005 27638974 PMC5724363

[37] Hedayati SS Yalamanchili V Finkelstein FO. A practical approach to the treatment of depression in patients with chronic kidney disease and end-stage renal disease. Kidney Int. (2011) 81:247–55. 10.1038/ki.2011.358 22012131 PMC3258342

[38] Beck A Steer R Brown G Van der Does A. BDI-II-NL Handleiding [BDI-II-Dutch Manual]. Lisse, The Netherlands: Psychological Corporation (2002).

[39] Little TD Cunningham WA Shahar G Widaman KF. To parcel or not to parcel: exploring the question, weighing the merits. Struct Equ Modeling. (2002) 9:151–73. 10.1207/S15328007SEM0902_1

[40] Wang XS Mendoza TR Gao SZ Cleeland CS. The Chinese version of the brief pain inventory (BPI-C): its development and use in a study of cancer pain. Pain. (1996) 67:407–16. 10.1016/0304-3959(96)03147-8 8951936

[41] LaRowe LR Farris SG Zvolensky MJ Ditre JW. Associations between past-month pain and distress intolerance among daily cigarette smokers. J Stud Alcohol Drugs. (2018) 79:781–9. 10.15288/jsad.2018.79.781 30422792 PMC6240006

[42] Hagger MS Orbell S. A meta-analytic review of the common-sense model of illness representations. Psychol Health. (2003) 18:141–84. 10.1080/088704403100081321

[43] Zhang N Fielding R Soong I Chan KK Lee C Ng A. Psychometric assessment of the Chinese version of the brief illness perception questionnaire in breast cancer survivors. PLoS ONE. (2017) 12:e0174093. 10.1371/journal.pone.0174093 28319160 PMC5358881

[44] Symister P Friend R. The influence of social support and problematic support on optimism and depression in chronic illness: a prospective study evaluating self-esteem as a mediator. Health Psychol. (2003) 22:123–129. 10.1037/0278-6133.22.2.123 12683732

[45] Fornell C Larcker DF. Evaluating structural equation models with unobservable variables and measurement error. J Mark Res. (1981) 18:39–50. 10.1177/002224378101800104

[46] Hu L-T Bentler PM. Evaluating Model Fit. Structural Equation Modeling: Concepts, Issues, and Applications. Thousand Oaks, CA: Sage Publications (1995). p. 76–99.

[47] Hancock GR Nevitt J. Bootstrapping and the identification of exogenous latent variables within structural equation models. Struct Equ Modeling. (1999) 6:394–9. 10.1080/10705519909540142

[48] Hair JF Black WC Babin BJ Anderson RE Tatham RL. Multivariate Data Analysis. vol. 5. Upper Saddle River, NJ: Prentice Hall (1998). p. 207–19.

[49] Furihata R Konno C Suzuki M Takahashi S Kaneita Y Ohida T. Unhealthy lifestyle factors and depressive symptoms: a Japanese general adult population survey. J Affect Disord. (2018) 234:156–61. 10.1016/j.jad.2018.02.093 29529548

[50] Köhler CA Freitas TH Maes M de Andrade NQ Liu CS Fernandes BS. Peripheral cytokine and chemokine alterations in depression: a meta-analysis of 82 studies. Acta Psychiatr Scand. (2017) 135:373–87. 10.1111/acps.12698 28122130

[51] Wang X Shen B Zhuang X Wang X Weng W. Investigating factors associated with depressive symptoms of chronic kidney diseases in China with type 2 diabetes. J Diabetes Res. (2017) 2017:1769897. 10.1155/2017/1769897 28261621 PMC5312451

[52] Hedayati SS Minhajuddin AT Toto RD Morris DW Rush AJ. Prevalence of major depressive episode in CKD. Am J Kidney Dis. (2009) 54:424–32. 10.1053/j.ajkd.2009.03.017 19493599 PMC3210064

[53] Chiang HH Livneh H Yen ML Li TC Tsai TY. Prevalence and correlates of depression among chronic kidney disease patients in Taiwan. BMC Nephrol. (2013) 14:78. 10.1186/1471-2369-14-78 23557031 PMC3626666

[54] Kroenke K Wu J Bair MJ Krebs EE Damush TM Tu W. Reciprocal relationship between pain and depression: a 12-month longitudinal analysis in primary care. J Pain. (2011) 12:964–973. 10.1016/j.jpain.2011.03.003 21680251 PMC3222454

[55] Reed C Hong J Novick D Lenox-Smith A Happich M. Health care costs before and after diagnosis of depression in patients with unexplained pain: a retrospective cohort study using the United Kingdom General Practice Research Database. Clinicoecon Outcomes Res. (2013) 5:37–47. 10.2147/ceor.s38323 23355787 PMC3552476

[56] Gerogianni G Kouzoupis A Grapsa E. A holistic approach to factors affecting depression in haemodialysis patients. Int Urol Nephrol. (2018) 50:1467–76. 10.1007/s11255-018-1891-0 29779116

[57] Choi NG Snow AL Kunik ME. Pain severity, interference, and prescription analgesic use among depressed, low-income homebound older adults. Aging Ment Health. (2016) 20:804–13. 10.1080/13607863.2015.1037244 25923452

[58] Cuff L Fann JR Bombardier CH Graves DE Kalpakjian CZ. Depression, pain intensity, and interference in acute spinal cord injury. Top Spinal Cord Inj Rehabil. (2014) 20:32–9. 10.1310/sci2001-32 24574820 PMC3919692

[59] Tang E Bansal A Novak M Mucsi I. Patient-reported outcomes in patients with chronic kidney disease and kidney transplant—Part 1. Front Med (Lausanne). (2017) 4:254. 10.3389/fmed.2017.00254 29379784 PMC5775264

[60] Burke AL Mathias JL Denson LA. Psychological functioning of people living with chronic pain: a meta-analytic review. Br J Clin Psychol. (2015) 54:345–360. 10.1111/bjc.12078 25772553

[61] Schaefert R Höner C Salm F Wirsching M Leonhart R Yang J. Psychological and behavioral variables associated with the somatic symptom severity of general hospital outpatients in China. Gen Hosp Psychiatry. (2013) 35:297–303. 10.1016/j.genhosppsych.2012.11.001 23219918

[62] Wilski M Tomczak M. Comparison of personal resources in patients who differently estimate the impact of multiple sclerosis. Ann Behav Med. (2017) 51:179–88. 10.1007/s12160-016-9841-5 27679463

[63] Jansen DL. Living with chronic kidney disease: the role of illness perceptions, treatment perceptions and social support (Dissertation). Utrecht University Repository, Utrecht, Netherlands (2012).

[64] Leventhal H Phillips LA Burns E. The common-sense model of self-regulation (CSM): a dynamic framework for understanding illness self-management. J Behav Med. (2016) 39:1–12. 10.1007/s10865-016-9782-2 27515801

[65] Hall RK Davenport CA Sims M Colón-Emeric C Washington T St. Clair Russell J. Association of functional and structural social support with chronic kidney disease among African Americans: the jackson heart study. BMC Nephrol. (2019) 20:262. 10.1186/s12882-019-1432-9 31307430 PMC6633656

[66] Darvishi A Otaghi M Mami S. The effectiveness of spiritual therapy on spiritual well-being, self-esteem and self-efficacy in patients on hemodialysis. J Relig Health. (2020) 59:277–88. 10.1007/s10943-018-00750-1 30673996

[67] Tawfic QA Bellingham G. Postoperative pain management in patients with chronic kidney disease. J Anaesthesiol Clin Pharmacol. (2015) 31:6–13. 10.4103/0970-9185.150518 25788766 PMC4353156

[68] Mehta S Peynenburg VA Hadjistavropoulos HD. Internet-delivered cognitive behaviour therapy for chronic health conditions: a systematic review and metaanalysis. J Behav Med. (2019) 42:169–87. 10.1007/s10865-018-9984-x 30387008

