# Supplementary material for: Depression and Associated Factors in Chinese Patients With Chronic Kidney Disease Without Dialysis: A Cross-Sectional Study
Source: Front Public Health. 2021 May 28;9:605651. doi: 10.3389/fpubh.2021.605651 (PMC8192721; doi:10.3389/fpubh.2021.605651)
Supplement: Supplementary file 1 [file Table_1.DOCX]

Appendix: Table A: Regression coefficient (β) of variables associated with depression scores from the univariate and multivariate linear regression models.

|  |  | Univariate lineal regression | | Multivariate lineal regression | |
| --- | --- | --- | --- | --- | --- |
| Characteristic |  | β | p value | β | p value |
| Sex | Male | Reference |  | Reference |  |
|  | Female | 0.214 | ＜0.001 | 0.081 | 0.265 |
| Age, (years) | ≤60 | Reference |  |  |  |
|  | ＞60 | -0.002 | 0.970 |  |  |
| Occupation | Employed | Reference |  | Reference |  |
|  | Unemployed | 0.159 | 0.004 | -0.081 | 0.344 |
| Education | College or above | Reference |  | Reference |  |
|  | Primary or below | 0.075 | 0.217 | 0.120 | 0.247 |
|  | Junior or high school | 0.196 | 0.001 | 0.166 | 0.129 |
| Marital status | Married | Reference |  |  |  |
|  | Single | 0.031 | 0.569 |  |  |
|  | Other | -0.02 | 0.721 |  |  |
| Place of residence | Urban | Reference |  |  |  |
|  | Suburban | 0.073 | 0.194 |  |  |
|  | Village | 0.046 | 0.411 |  |  |
| Monthly personal income (RMB) | <2000 or no income | Reference |  | Reference |  |
|  | 2000–5000 | -0.249 | ＜0.001 | 0.039 | 0.673 |
|  | 5000– 8000 | -0.275 | ＜0.001 | 0.161 | 0.107 |
| Smoking | Current smoker | Reference |  |  |  |
|  | Ex-smoker | 0.028 | 0.712 |  |  |
|  | Never smoker | 0.071 | 0.346 |  |  |
| Alcohol drinking | Current drinker | Reference |  |  |  |
|  | Ex-drinker | -0.026 | 0.644 |  |  |
|  | Never drinker | -0.032 | 0.562 |  |  |
| Months since CKD diagnosis | ≤12 | Reference |  |  |  |
|  | 12 ~ 60 | -0.06 | 0.320 |  |  |
|  | 60 ~ 120 | 0.04 | 0.506 |  |  |
|  | ＞ 120 | -0.06 | 0.295 |  |  |
| CKD stage | G1 | Reference |  |  |  |
|  | G2 | -0.033 | 0.622 |  |  |
|  | G3 | 0.022 | 0.752 |  |  |
|  | G4 | 0.103 | 0.111 |  |  |
|  | G5 | 0.099 | 0.100 |  |  |
| Comorbidity | Hypertension | Reference |  |  |  |
|  | Diabetes | 0.031 | 0.594 |  |  |
|  | Both | -0.043 | 0.457 |  |  |
|  | Other | -0.001 | 0.991 |  |  |
|  | None | -0.048 | 0.445 |  |  |
| RESE scores |  | -0.468 | ＜0.001 | -0.280 | 0.002 |
| BIPQ scores |  | 0.535 | ＜0.001 | 0.337 | 0.001 |
| PI scores |  | 0.459 | ＜0.001 | 0.323 | ＜0.001 |

Note: RESE: Rosenberg Self-Esteem Scale; BIPQ: Brief Illness Perception Questionnaire; PI: Pain interference
